# Features Discriminating COVID-19 From Community-Acquired Pneumonia in Pediatric Patients

**DOI:** 10.3389/fped.2020.602083

**Published:** 2020-11-05

**Authors:** Yu Guo, Wei Xia, Xuehua Peng, Jianbo Shao

**Affiliations:** Department of Imaging Center, Wuhan Children's Hospital (Wuhan Maternal and Child Healthcare Hospital), Tongji Medical College, Huazhong University of Science and Technology, Wuhan, China

**Keywords:** COVID-19, children, computed tomography, Community-Acquired Pneumonia (CAP), respiratory infection

## Abstract

**Purpose:** To discuss the different characteristics of clinical, laboratory and chest computed tomography (CT) between coronavirus disease 2019 (COVID-19) and community-acquired pneumonia (CAP) in pediatric patients.

**Methods:** We retrospectively retrieved data of inpatients with COVID-19 from January 21st to March 14th, 2020, and CAP from November 1st, 2019 to December 31st, 2019 in Wuhan Children's Hospital. We divided CAP into mycoplasma pneumonia and other viral pneumonia. We analyzed clinical and radiological features from those patients, and compared the differences among COVID-19, mycoplasma pneumonia and other viral pneumonia.

**Results:** Eighty COVID-19 inpatients from January 21st to March 14th, 2020, as well as 95 inpatients with mycoplasma pneumonia and 50 inpatients with other viral pneumonia from November 1st, 2019 to December 31st, 2019 were included in our study. All patients were confirmed with RT-PCR. The clinical symptoms were similar in the three groups. Except fever and cough, diarrhea (6/80, 7.5%), tachypnea (2/80, 2.5%), and fatigue (6/80, 7.5%) were less common in COVID-19 patients. Compared to mycoplasma pneumonia and other viral pneumonia inpatients, COVID-19 patients present remarkably increased alanine aminotransferase (69/80, 86.3%). The typical CT feature of COVID-19 is ground-glass opacity, and it was more common in COVID-19 patients (32/80, 40%).

**Conclusion:** The COVID-19 shared similar onsets with CAP. Even though the ground-glass opacity and elevated level of ALT were frequent in COVID-19, the better way for treatment and management of this disease is quickly and accurately identifying the pathogen.

## Introduction

In late December 2019, the pneumonia caused by a novel coronavirus (SARS-Cov-2) was identified in Wuhan Hubei province, China (1). The world health organization named the disease caused by SARS-Cov-2 coronavirus disease 2019 (COVID-19) (2). By September 1st, 2020, 90,383 confirmed cases of COVID-19 in China, and 25,118,689 cases in 216 countries (3). Person-to-person transmission of SARS-CoV-2 occurs through close contact with infected person, mainly via respiratory droplets and after touching contaminated objects (4). And the infections showed familiar aggregation, pediatric patients infected by SARS-CoV-2 appeared later than adult (5, 6). At present, defining the clinical characteristics of the disease in large cohorts of patients is an urgent need. While plenty of data are available for adult patients with COVID-19, limited reports were available for pediatric patients infected with SARS-CoV-2 (5–7).

Pneumonia is the most common cause of illness in children (8). Community-acquired pneumonia accounts for a large part of pediatric pneumonia. Many pathogens, including bacteria, viruses and other microorganisms, are associated with pneumonia. And viral pneumonia and mycoplasma pneumonia in children are common during autumn and winter (9). So it is very hard time for public health and doctors in this outbreak.

Mycoplasma pneumoniae (MP) is one of most important etiologic agents causing community-acquired pneumonia (CAP) in children (10, 11). The common pathogens of pediatric viral pneumonia include respiratory syncytial virus, influenza A/B virus and so on (11–13). The clinical symptoms of COVID-19 are similar to the other pediatric pneumonias, but there were effective treatments for pneumonia caused by these pathogens already (11). So it is important to distinguish COVID-19 from other pediatric pneumonias.

The aim of this study was to compare clinical, laboratory and radiological results of COVID-19 patients collected during the COVID-19 outbreak with other pneumonia patients collected before COVID-19 outbreak in the same hospital. If early discriminating features are recognized, they may facilitate the diagnosis of COVID-19 and timely control the transmission of COVID-19, since the overall detection rate for SARS-CoV-2 RNA by PCR assays is currently only 60% in the 1st week of illness. It would be important to find the differences in distinguishing SARS from other respiratory illnesses.

## Methods

### Patients

For comparative study, we extracted data of 109 COVID-19 inpatients from January 21st to March 14th, 2020 and 166 non-COVID-19 inpatients from November 1st, 2019 to December 31st, 2019, before this outbreak, at Wuhan Children's Hospital. Among 109 COVID-19 inpatients, 26 patients had COVID-19 co-infection with mycoplasma pneumoniae, three patients had COVID-19 co-infection with other viruses. Among 166 non-COVID-19 inpatients, 50 patients infected by other virus (influenza A virus, influenza B virus, respiratory syncytial virus, parainfluenza virus and adenovirus) and 95 patients infected by mycoplasma pneumoniae, 18 patients had mycoplasma pneumoniae co-infection with other viruses, three patients infected with multi-viruses. Excluded all co-infected patients, 80 COVID-19 patients, 50 patients infected by only one type of virus and 95 patients infected by mycoplasma pneumoniae were included in our study. We defined patients infected with mycoplasma pneumoniae as mycoplasma pneumonia patients, and defined patients infected with only one type of virus as other viral pneumonia patients.

### Inclusion and Exclusion Criteria

Patients were included in the study if they met the following criteria: (a) presented to the emergency department and required hospitalization; (b) had a respiratory illness, as defined by the presence of lower respiratory symptoms (cough, sputum production, shortness of breath); (c) performed a chest CT scan; and (d) had positive etiological confirmation and identified a single pathogen infection. Patients without established etiologies or co-infected were excluded.

### Identification of Pathogens

According to the guideline of laboratory detection for COVID-19 (14), we collected throat swabs or/and sputa from suspected patients to detect the pathogen by RT-PCR. Serological assays were performed to detect mycoplasma pneumoniae by detecting IgM and Respiratory Virus Identification Kit (D3 Ultra TM DFA, Diagnostic Hybrids) was used to detect and identify influenza A virus, influenza B virus, respiratory syncytial virus, parainfluenza virus, adenovirus.

### Data Collection

We reviewed clinical chart, laboratory findings and chest CTs for all COVID-19 and non-COVID-19 patients. The admission data of these patients were from Nov 1st to Dec 31st, 2019 and January 21st to March 14th, 2020. For all patients, non-contrast chest CT studies were performed on SMATON Definiton AS128 unit (Siemens, Siemens medical system, Germany). All the images were reviewed by two experienced pediatric radiologists.

### Statistical Analysis

Demographic, clinical, laboratory and radiological features were analyzed using Chi-square test or Fisher's exact test for categorical variables. Continuous variables were expressed as mean ± SD and compared with the Mann-Whitney *U*-test. Data were analyzed using SPSS version 25. All analyses were two tailed and a *p*-value of < 0.05 was considered statistically significant.

## Result

**Figure 1:**
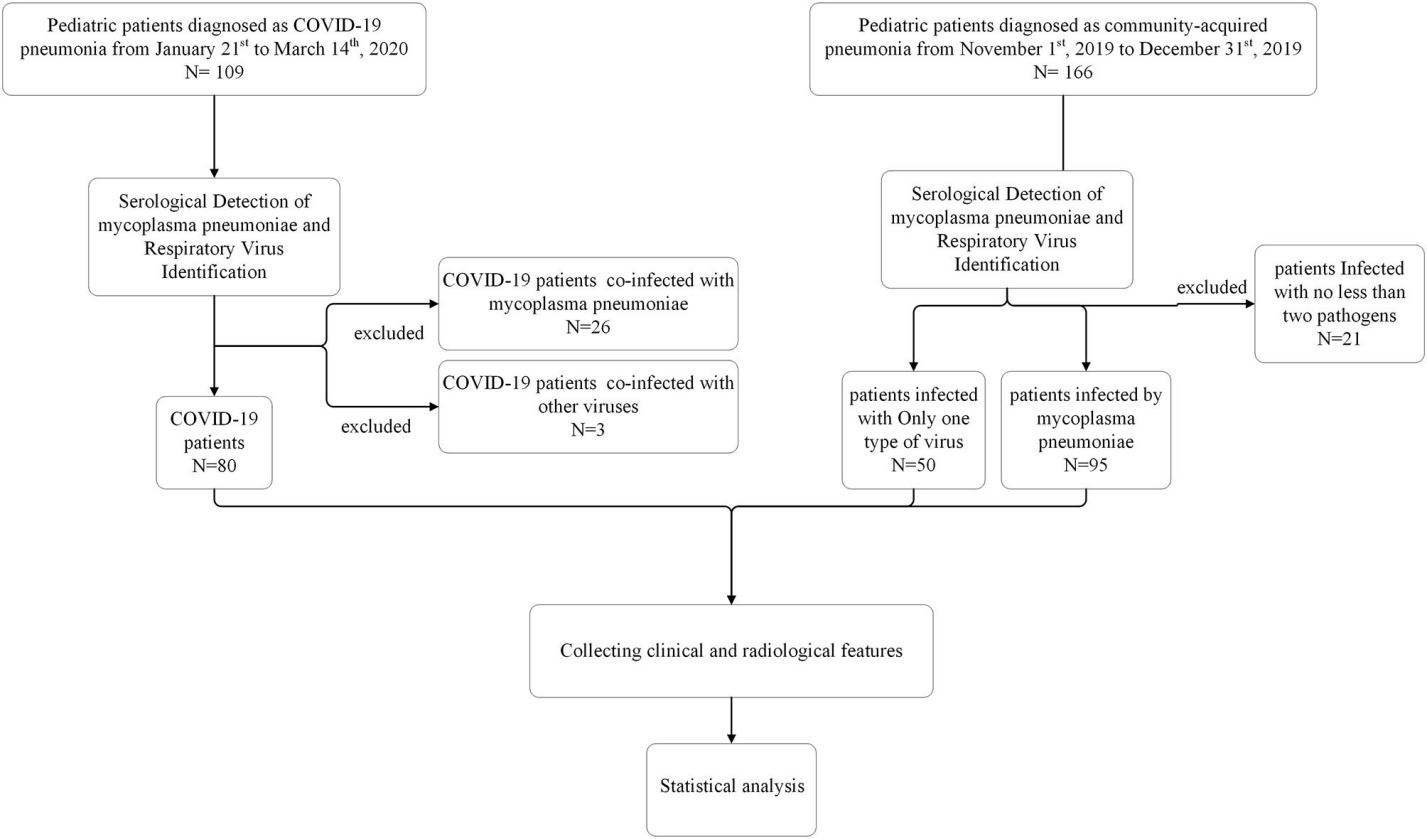
Flow chart for patient selection.

We reported a comparative analysis on 80 pneumonia patients with SARS-CoV-2 infection, 95 pneumonia patient with mycoplasma pneumoniae infection and 50 pneumonia patient with other virus infection. Other viral pneumonia patients consisted of 35 patients infected with respiratory syncytial virus, one patient infected with influenza A, six patients infected with influenza B, six patients infected with parainfluenza virus and two patients infected with adenovirus. All patients are identified as a single pathogen infection (Figure 1).

### Clinical Features of Cases

The demographic characteristics of pediatric COVID-19 patients and non-COVID-19 patients were displayed in Table 1. In this study, patient ages ranged 18 days to 15 years old, the mean age was 5.99 ± 4.77 in COVID-19 patients, 5.03 ± 3.26 in mycoplasma pneumoniae patients and 1.03 ± 1.38 in other viral pneumonia patients. COVID-19 patients and mycoplasma pneumonia patients were mainly distributed in the group of over 6 years old (48.8 and 48.4%), while other viral pneumonia patients were concentrated in the group of under 1 year old (68%), and the proportion of population in the COVID-19 patients and other viral pneumonia patients is significantly different between the group under 1 year old (*p* < 0.0001) and the group over 6 years old (*p* < 0.0001). Twenty eight (35%) were female in COVID-19 patients, 59 (62.1%) in mycoplasma pneumoniae patients, and 14 (28%) in other viral pneumonia patients. On admission, the most common symptoms were fever and cough in all patients, COVID-19 (38/80 [47.5%] and 39/80 [48.4%]), mycoplasma pneumoniae (77/95 [81.1%] and 83/95 [87.4%]), other viral pneumonia (25/50 [50%] and 48/50 [96%]). Less common symptoms in COVID-19 patients were diarrhea (6/80, 7.5%), tachypnea (2/80, 2.5%) and fatigue (6/80, 7.5%). These symptoms were less common in mycoplasma pneumoniae patients too, but proportion of tachypnea in other viral pneumonia patients (30/50, 60%) was higher than other groups. In comparison, no significant differences were observed between COVID-19 and mycoplasma pneumoniae patients on these symptoms, but there were significant differences between COVID-19 and other viral pneumonia patients in age distribution of onset and some symptoms.

**Table 1:** The Clinical features of pediatric patients.

**Characteristic**	**COVID-19 *N* = 80**	**Mycoplasma*N* = 95**	**Other virus *N* = 50**	***p*^a^**	***p*^b^**
Sex					
Boy	52 (65%)	36 (37.9%)	36 (72%)	NULL	NULL
Girl	28 (35%)	59 (62.1%)	14 (28%)	NULL	NULL
Age					
<1 y	18 (22.5%)	9 (9.5%)	34 (68%)	0.017	<0.0001
1–3 y	12 (15%)	14 (14.7%)	9 (18%)	0.961	0.651
3–6 y	11 (13.8%)	26 (27.4%)	6 (12%)	0.028	0.773
>6 y	39 (48.8%)	46 (48.2%)	1 (2%)	0.965	<0.0001
Symptom					
Fever	38 (47.5%)	77 (81.1%)	25 (50%)	<0.0001	0.781
Cough	39 (48.8%)	83 (87.4%)	48 (96%)	<0.0001	<0.0001
Diarrhea	6 (7.5%)	2 (2.1%)	6 (12%)	0.089	0.388
Tachypnea	2 (2.5%)	12 (12.6%)	30 (60%)	0.014	<0.0001
Fatigue	6 (7.5%)	7 (7.4%)	12 (24%)	0.974	0.008

a *Group COVID-19 vs. Group mycoplasma pneumonia*.

b *Group COVID-19 vs. Group other viral pneumonia*.

*NULL, not appropriate for statistical analysis*.

### Laboratory Findings of the Patients

Table 2 shows findings of laboratory examinations related to immunological responses and cardiac, liver damage. There were significant differences between COVID-19 patients and mycoplasma pneumonia patients in many laboratory findings except for procalcitonin (PCT) and Creatine kinase-MB (CK-MB). WBC count of most COVID-19 patients (7.67 ± 3.08, ^*^10^9^/L) were less than mycoplasma pneumonia patients (11.58 ± 6.78, ^*^10^9^/L) (*p* < 0.0001). The level of C-reaction protein (CRP) in mycoplasma pneumonia patients (1.22 ± 7.16) was higher than COVID-19 patients (4.21 ± 8.97) (*p* < 0.0001). While lymphocytes and alanine aminotransferase (ALT) in COVID-19 patients were higher than mycoplasma pneumonia patients. The laboratory characteristics of COVID-19 patients are similar to those of other viral pneumonia patients. It was worth noting that the level of CRP in COVID-19 patients (4.21 ± 8.97) was lower than other viral pneumonia patients (9.85 ± 16.77) (*p* = 0.032). And ALT of COVID-19 patients was highest in three group. As the reference range were different for WBC, lymph in different age groups, we had to categorize them as elevated, normal or decreased one according to the reference values (15–17). We represented it in Supplementary Table. The laboratory findings of COVID-19 pneumonia and other viral pneumonia were similar, except decreased ratio of lymphocytes (*p* < 0.0001), elevated PCT (*p* < 0.0001) and elevated ALT (*p* < 0.0001). There were significant differences between COVID-19 patients and mycoplasma pneumonia patients in many laboratory findings except for CK-MB. WBC count of most COVID-19 patients were in normal range (66/80, 82.5%), while that was not common in mycoplasma pneumonia patients (45/95, 47.4%) (*p* < 0.0001). Decreased ratio of lymphocytes in mycoplasma pneumonia patients (79/95, 83.2%) was more common than COVID-19 (14/80, 17.5%) (*p* < 0.0001), and elevated levels of CRP in mycoplasma pneumonia patients (66/95, 69.5%) was also more common than COVID-19 (20/80, 25%) (*p* < 0.0001). Comparison of three groups, elevated ALT (69/80, 86.3%) (*p* < 0.0001) was more common in COVID-19 than non-COVID-19. This reminded us that COVID-19 patients would suffer liver injury, even though most COVID-19 patients presented with mild symptoms. According to our findings, it was easy to distinguish COVID-19 and mycoplasma pneumonia in laboratory examinations, while laboratory findings of COVID-19 were similar to other viral pneumonia. It was noteworthy that the level of ALT in most COVID-19 patients were abnormal (69/80, 86.3%), and we should pay more attention to liver function in treatment.

**Table 2:** Laboratory features of pediatric patients.

	**COVID-19 *N* = 80**	**Mycoplasma*N* = 95**	**Other virus *N* = 50**	***p*^a^**	***p*^b^**
WBC, *10^9^/L	7.67 ± 3.08	11.58 ± 6.78	8.63 ± 3.04	<0.0001	0.085
LYM%	48.37 ± 12.13	28.71 ± 16.50	46.96 ± 18.44	<0.0001	0.669
CRP, mg/L	4.21 ± 8.97	19.40 ± 33.72	9.85 ± 16.77	<0.0001	0.032
PCT, mg/L	0.10 ± 0.14	1.22 ± 7.16	0.37 ± 1.11	0.131	0.089
ALT, U/L	45.66 ± 19.37	17.29 ± 19.30	31.44 ± 37.19	<0.0001	0.005
CK-MB, U/L	34.13 ± 29.06	37.78 ± 33.64	45.04 ± 30.07	0.448	0.042

a *Group COVID-19 vs. Group mycoplasma pneumonia*.

b *Group COVID-19 vs. Group other viral pneumonia*.

### The Features of CT Images

CT imaging findings were shown in Table 3. Although COVID-19 patients were confirmed by RT-PCR tests, only 56 (70%) COVID-19 cases had definite pneumonia visible on chest CTs, while all patients infected by mycoplasma (*p* < 0.0001) or other virus (*p* < 0.0001) had abnormal on chest CT. There were more patients presented with unilateral pulmonary lesions in COVID-19 (34/80, 42.5%), while there were more inclined to bilateral lesions in other viral pneumonia patients (46/50, 92%) (*p* < 0.0001). The lesions were more concentrated in the lower lobes in all patients. The typical feature of COVID-19 is ground-glass opacity (6), and it presented more in COVID-19 patients (32/80, 40%) than mycoplasma pneumonia (5/95, 5.3%) (*p* < 0.0001) and other viral pneumonia patients (1/50, 2%) (*p* < 0.0001). The other typical feature is consolidation (6), while it was more common in mycoplasma pneumonia (93/95, 97.9%) (*p* < 0.0001) and other viral pneumonia patients (49/50, 98%) (*p* < 0.0001) than COVID-19 patients (31/80, 38.8%). In the three groups, the streaky sign was not as obvious in COVID-19 patients (3/80, 3.8%) as mycoplasma pneumonia (14/95, 14.7%) (*p* = 0.014) and other viral pneumonia patients (7/50, 14%) (*p* = 0.033).

**Table 3:** CT imaging findings in pediatric patients.

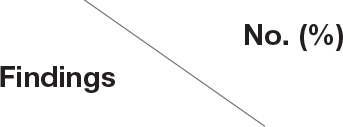	**COVID-19 *N* = 80**	**Mycoplasma *N* = 95**	**Other virus *N* = 50**	***p*^a^**	***p*^b^**
Pulmonary lesions					
Null	24 (30%)	0 (0%)	0 (0%)	<0.0001	<0.0001
Unilateral pneumonia	34 (42.5%)	45 (47.4%)	14 (28%)	0.519	0.096
Bilateral pneumonia	22 (27.5%)	50 (52.6%)	36 (72%)	0.001	<0.0001
Distribution					
Left upper lobe	16 (20%)	48 (50.5%)	43 (86%)	<0.0001	<0.0001
Left lower lob	29 (36.3%)	56 (58.9%)	46 (92%)	0.003	<0.0001
Right upper lobe	20 (25%)	46 (48.4%)	39 (78%)	0.001	<0.0001
Right middle lobe	16 (20%)	48 (50.5%)	40 (80%)	<0.0001	<0.0001
Right lower lobe	30 (37.5%)	58 (61.1%)	44 (88%)	0.002	<0.0001
Manifestation					
Consolidation	31 (38.8%)	93 (97.9%)	49 (98%)	<0.0001	<0.0001
Cord sign	3 (3.8%)	14 (14.7%)	7 (14%)	0.014	0.033
Ground-glass opacities	32 (40%)	5 (5.3%)	1 (2%)	<0.0001	<0.0001

a *Group COVID-19 vs. Group mycoplasma pneumonia*.

b *Group COVID-19 vs. Group other viral pneumonia*.

## Discussion

Until now, COVID-19 still poses a huge threat to human survival. More and more children are infected all over the world, it is important to understanding the manifestations of COVID-19 in children. Pneumonia is the most common disease in children, understanding the clinical features of COVID-19 in pediatric patients and distinguishing CAP in children are important for diagnosis and effective treatment.

In our study, the prevalence of COVID-19 patients and other viral pneumonia patients is significantly different between the group under 1 year old and the group over 6 years old (Table 1). School-age children are more susceptible to COVID-19. Since the outbreak of COVID-19 in the winter vacation, school-age children have their own small social circle, they would be exposed to more potential patients, so the prevalence of COVID-19 in this age group was higher than other viral pneumonia. The clinical symptoms were similar in three groups. Fever and cough were common in cases with COVID-19, mycoplasma pneumonia or other viral pneumonia. As COVID-19 was mostly mild in children, tachypnea (2.5%) and fatigue (7.5%) were less common in COVID-19. The similar symptoms make it hard to distinguish the pathogens, as well as control this epidemic. So it is very important to identify the pathogen.

As the symptoms were similar in COVID-19 and CAP, we analyzed laboratory and radiological features. A striking characteristic of COVID-19 differs from CAP is that it affects the function of liver, as shown by elevated amounts of alanine aminotransferase (86.3%), even though all children with COVID-19 was mild or moderate according to clinical type. Previous reports showed that adult patients with COVID-19 had different degrees of liver function abnormality (18, 19), but it was infrequent in pediatric patients. Elevated level of ALT may be important for subsequent treatment planning.

**Figure 2:**
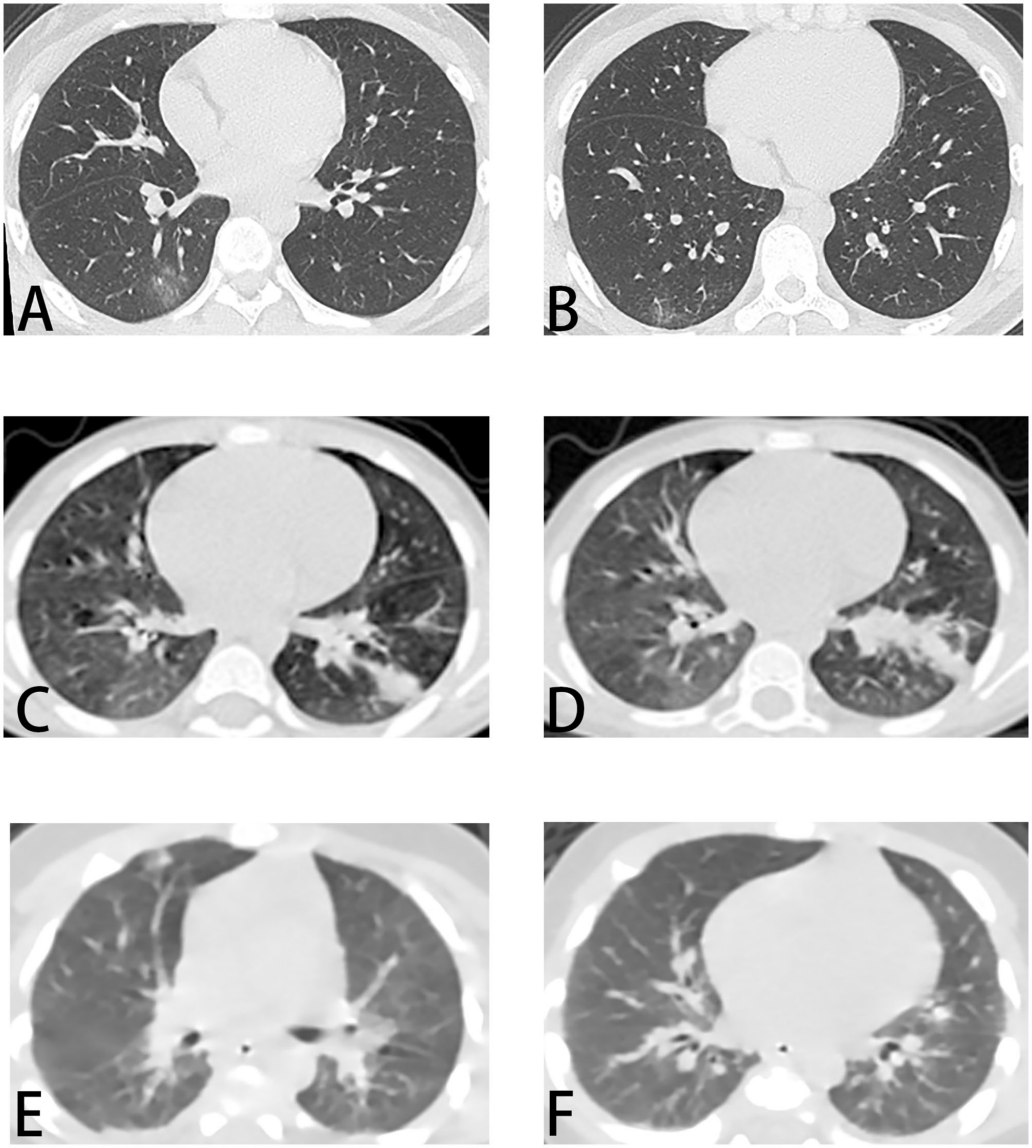
Chest CT images of patients. **(A,B)** COVID-19. Male, 15 years old. On admission, chest CT showed ground-glass opacities in the inferior lobe of the right lung. **(C,D)** Mycoplasma pneumonia. Male, 4 years old. Chest CT showed bilateral lesions with consolidation in inferior lobe of the left lung. **(E,F)** Other viral pneumonia (respiratory syncytial virus). Male, 1 month old. Chest CT showed diffused consolidations in both lungs. CT, computed tomography.

Because clinical signs and symptoms are poor predictors for pediatric pulmonary infections, and chest radiography is not sensitive enough, chest CT will be helpful with diagnosis. Our study of chest CTs in COVID-19 in children showed the lung lesions were mainly concentrated in the lower lobes, while lesions of mycoplasma pneumonia and other viral pneumonia patients are more diffuse (Figure 2, Table 3). And the ground-glass opacity (40%) and consolidation (38.8%) were typical manifestations in COVID-19 pediatric patients (6), similar to those in adults (20). And the ground-glass opacity was rare in mycoplasma pneumonia (5.3%) and other viral pneumonia (2%). Even though the ground-glass opacity can be used as a typical sign, there were 24 (30%) COVID-19 patients showed normal on chest CT.

Above all, it is difficult to distinguish COVID-19 and CAP in children from these clinical features. As a high morbidity of COVID-19, and at this time there are no specific vaccines or treatments, establish a method to quickly and accurately identify the pathogen is a reasonable strategy and is likely to be more effective in treatment and management.

Nevertheless, the present study had several limitations. It was conducted at a single center, the applicability of our findings may vary depending upon the relative prevalence of the etiological agent during any particular period. And the non-COVID-19 groups are comprised of relatively severe pneumonia patients. The non-COVID-19 patients with mild pneumonia were excluded. There was a selection bias in the non-COVID-19 group. There were limitations of our study.

In conclusion, the results of this study indicated COVID-19 has similar onsets with mycoplasma pneumonia and other viral pneumonia, while it appears to have a mild or moderate course in pediatric patients. And there was less fever, cough and tachypnea in the COVID-19 patients than CAP patient. Remarkably, liver function damage is more frequent in COVID-19 than CAP patients. On chest CTs, the ground-glass opacity is the typical feature. In this study, we compared COVID-19 and other common pneumonia in pediatric patients, and we suggest to consider the clinical features, laboratory results and CT findings together on pediatric patients with COVID-19. Combining with pathogen identification, reasonable management could be drawn out under this circumstance.

## Data Availability Statement

The original contributions generated for this study are included in the article/Supplementary Material, further inquiries can be directed to the corresponding author/s.

## Ethics Statement

This study was approved by the Research Ethics Board of Wuhan Children's Hospital (No. 2020208).

## Author Contributions

WX and JS designed the study. YG collected patient's data, analyzed data, prepared tables and figures, and wrote the manuscript. JS supervised data collection. JS, WX, and XP reviewed and revised the manuscript. All authors contributed to the article and approved the submitted version.

## Conflict of Interest

The authors declare that the research was conducted in the absence of any commercial or financial relationships that could be construed as no potential conflict of interest.

## Abbreviations

COVID-19: coronavirus disease 2019
CT: computed tomography
RT-PCR: reverse transcription-polymerase chain reaction
SARS-CoV-2: severe acute respiratory syndrome coronavirus 2
PCT: procalcitonin
ALT: alanine aminotransferase.
