# Supplementary material for: Features Discriminating COVID-19 From Community-Acquired Pneumonia in Pediatric Patients
Source: Front Pediatr. 2020 Nov 5;8:602083. doi: 10.3389/fped.2020.602083 (PMC7676900; doi:10.3389/fped.2020.602083)
Supplement: Supplementary file 1 [file Table_1.DOCX]

Supplementary Table Laboratory information of pediatric patients

|  | **COVID-19**  **N=80** | **Mycoplasma**  **N=95** | **Other virus**  **N=50** | ***p*^a^** | ***p*^b^** |
| --- | --- | --- | --- | --- | --- |
| **WBC, *10^9^/L** |  |  |  |  |  |
| **decreased** | 2(2.5%) | 15(15.8%) | 8(16%) | 0.003 | 0.013 |
| **normal** | 66(82.5%) | 45(47.4%) | 34(68%) | <0.0001 | 0.056 |
| **elevated** | 12(15%) | 35(36.8%) | 8(16%) | 0.001 | 0.878 |
| **LYM%** |  |  |  |  |  |
| **decreased** | 14(17.5%) | 79(83.2%) | 24(48%) | <0.0001 | <0.0001 |
| **normal** | 42(52.5%) | 14(14.7%) | 17(34%) | <0.0001 | 0.039 |
| **elevated** | 24(30%) | 2(2.1%) | 9(18%) | <0.0001 | 0.126 |
| **CRP, mg/L** |  |  |  |  |  |
| **normal** | 60(75%) | 29(30.5%) | 30(60%) | <0.0001 | 0.071 |
| **elevated** | 20(25%) | 66(69.5%) | 20(40%) | <0.0001 | 0.071 |
| **PCT, mg/L** |  |  |  |  |  |
| **normal** | 36(45%) | 14(14.7%) | 0(0%) | <0.0001 | <0.0001 |
| **elevated** | 44(55%) | 81(85.3%) | 50(100%) | <0.0001 | <0.0001 |
| **ALT, U/L** |  |  |  |  |  |
| **normal** | 11(13.8%) | 89(93.7%) | 43(86%) | <0.0001 | <0.0001 |
| **elevated** | 69(86.3%) | 6(6.3%) | 7(14%) | <0.0001 | <0.0001 |
| **CK-MB, U/L** |  |  |  |  |  |
| **normal** | 40(50%) | 46(48.4%) | 12(24%) | 0.835 | 0.003 |
| **elevated** | 40(50%) | 49(51.6%) | 38(76%) | 0.835 | 0.003 |

a, Group COVID-19 *vs* Group mycoplasma pneumonia;

b, Group COVID-19 *vs* Group other viral pneumonia;

WBC, white blood cell; LYM, lymphocyte; CRP, C-reactive protein; PCT, procalcitonin; ALT, alanine aminotransferase; CK-MB, Creatine kinase-MB

Normal reference (hospital-self-defined values)^1-3^

WBC (*10^9^/L), 5.3-12.5 (< 1 year old), 4.4-11.1 (1-3 years old), 4.4-10.9 (3-6 years old), 4.0-10.0 (> 6 years old); LYM%, 45-60 (< 1 year old), 28-65 (1-6 years old), 26-58 (> 6 years old); CRP (mg/L), 0-3; PCT (mg/L), 0-0.05; ALT (U/L), 0-40; CK-MB (U/L), 0-25.

Reference

[1] Gao y, Zou C, Jiang J, Yang J, Tian X. [Determination of normal reference ranges for venous blood count among 526 children aging from 1year old to 12 years old in Shanghai]. Guo Ji Jian Yan Yi Xue Za Zhi. 2015;16:2332-4.

[2] Du Y, Wang W, He F, Zhong K, Yuan S, Liu J, et al. [Investigation and analysis on pediatric reference intervals of routine chemistry items in China]. Jian Yan Yi Xue. 2020;7:676-81.

[3] Zhong X, Ding J, Zhou J, Yu Z, Sun S, Bao Y, et al. [A multicenter study of reference intervals for 15 laboratory parameters in Chinese children]. Zhonghua Er Ke Za Zhi. 2018;56:835-45.
